# Assessing the effectiveness of an antiracism clinical skills curriculum for medical students: a single institution mixed methods study

**DOI:** 10.1186/s12909-026-08854-z

**Published:** 2026-02-21

**Authors:** Rohini Jain, Leanna Lewis, Katherine Brooks, Ashkon Shaahinfar, Shelene Stine, Jyothi Marbin, Odinakachukwu Ehie

**Affiliations:** 1 Department of Pediatrics, University of California, San Francisco Benioff Children’s Hospital, San Francisco, CA USA; 2 Division of Pediatric Oncology, University of California, San Francisco Benioff Children’s Hospital, San Francisco, CA USA; 3 Joint Medical Program (JMP), UC Berkeley-UCSF, Berkeley, CA USA; 4 Division of Hospital Medicine, Zuckerberg San Francisco General Hospital, San Francisco, CA USA; 5 Department of Emergency Medicine, University of California, San Francisco, San Francisco, CA USA; 6 Department of Anesthesia and Perioperative Services, University of California, San Francisco Benioff Children’s Hospital, San Francisco, CA USA

**Keywords:** Antiracism, Healthcare disparities, Curriculum development, Undergraduate medical education, Clinical skills

## Abstract

**Background:**

Despite knowledge that systemic racism exists in medicine and increased efforts within medical education to address this, educators continue to struggle with incorporating training for future healthcare providers on how to actively address implicit bias and structural patterns of racism into formalized curricula. This study sought to understand whether an antiracism clinical skills curriculum (ARC) for pre-clinical medical students (MS) at the UC Berkeley-UCSF Joint Medical Program (JMP) improved their ability to recognize how structural racism can influence clinical encounters and begin to identify tools to counteract this.

**Methods:**

The ARC was developed by core faculty at the JMP using Kern’s six-step approach to curriculum development and then taught to a cohort of 32 pre-clinical MS over the 2022–2023 academic year. To evaluate the effectiveness of the ARC, we utilized the Kirkpatrick model and a mixed methods approach with pre-/post-surveys as well as semi-structured individual interviews to evaluate students’ reactions and learning. Instructional methods included standardized patients, journal article review, reflective exercises, small group case discussions, didactic lectures, and community member testimonies.

**Results:**

Overall, 28/32 (87.5%) of students participated in the study, with 20/32 (62.5%) completing both the pre- and post-surveys. Of those participating, 13/28 (46.4%) agreed to complete an individual interview. After completing the ARC, students reported more familiarity with 13/14 (92.9%) learning objectives, including recognizing how patients experience racism in the medical setting. Prior to the ARC, students had a mean score of 2.9 on a 5-point Likert Scale (1 = not at all familiar and 5 = very familiar) related to their knowledge of race-based medicine and after the ARC, students had a mean score of 4.55 (*p* < 0.05). A major theme that emerged from the interviews was a desire for education in this area to shift from raising awareness to a more solution-oriented focus.

**Conclusions:**

These results demonstrate that while many MS are aware of health disparities, few can identify practical tools to help tackle these disparities. Even though most MS in this study believe antiracism training is important, many fear that this type of curriculum is deprioritized within an educational system that lacks institutional and structural support.

**Supplementary Information:**

The online version contains supplementary material available at 10.1186/s12909-026-08854-z.

## Background

Systemic racism and bias continue to influence how healthcare is utilized and delivered. There are many examples of how this manifests, including differential assessment of pain between White and Black patients, the overdiagnosis of cardiac symptoms in women as anxiety, and differential care based on insurance status [1]. By failing to address these underlying factors, we are continuing to further disadvantage the health of already marginalized communities [2]. Despite increasing interest among medical educators in how systemic racism impacts health outcomes, there is still varied practice on how educators incorporate training related to actively addressing implicit bias and structural patterns of racism into formalized curricula [3, 4].

In the current political climate, where diversity, equity, and inclusion (DEI) programs are under ongoing threat of being dismantled by legislative, judicial, and executive branch decisions, it is time to focus on expanding the traditional medical education curriculum to teach students to become active participants in addressing discrimination in medicine. In 2020, the Association of American Medical Colleges (AAMC) released a framework designed to guide the medical community to begin addressing structural racism within medicine, focusing on pillars such as individual self-reflection and anti-racism efforts within both the academic medical community and broader community organizations [5]. This ignited a spark for leaders of the medical education community to develop strategies for what an effective antiracism curriculum (ARC) should look like and how to best implement it [6, 7]. Core ideas to consider when structuring an ARC include: (1) raising awareness at an institutional level, (2) engaging topic experts and fostering student engagement, and (3) establishing metrics to measure progress in antiracism work [8]. A recent cross-sectional study to evaluate the current state of pedagogy on antiracism in US Academic Health Centers similarly found that inclusion of antiracism in medical education requires intentional training and changes at the institutional level (Fatahi, [4]). Many initiatives sought to incorporate antiracism topics as part of a larger health equity curriculum, with dedicated seminars or small group sessions [9, 10]. However, it soon became clear that brief or elective trainings focused on antiracism were not the solution, and there needed to be a shift towards longitudinal integration of these topics into the medical education curriculum as a whole [11]. Additionally, up to this point, the majority of ARC have been ancillary and separated from the clinical context of medical education. Furthermore, to be truly successful, antiracist efforts must be prioritized at both structural and institutional levels. One proposed step towards doing this is including antiracist metrics (i.e. the number of underrepresented faculty promoted and retained at each academic rank) within accreditation standards so that institutions are motivated to be accountable [12].

With this in mind, faculty at the UC Berkeley-UCSF Joint Medical Program (JMP) developed an innovative ARC using Kern’s six-step approach to curriculum development that ties in antiracism learning objectives within clinical skills competency goals during a longitudinal, spiraling curriculum [13]. This framework allows students to revisit key concepts over time and build on prior knowledge. The ARC blends case discussions with simulated patient encounters and affinity group caucusing to create a truly integrated environment for students to engage with antiracism work. Affinity group caucusing provides opportunities for differentiated learning and promotes critical introspection and deeper conversations, with increased psychological safety, further building community among students [14]. This study sought to understand whether an ARC that is interwoven with clinical skills improved the ability of pre-clinical medical students at the JMP to recognize how structural racism can influence clinical encounters and begin to identify tools to counteract this.

## Methods

### Curriculum design

The mission of the UC Berkeley-UCSF JMP is to “develop outstanding physicians and collaborative changemakers with the skills to solve public health and health equity challenges and improve the wellbeing of patients and communities.” The clinical skills curriculum in each year was delivered over 36 weekly 3-hour clinical skills sessions (18 in the Fall and 18 in the Spring), with each session having associated antiracism connections and resources. Additionally, there are two sessions in the second-year curriculum that are dedicated to “Mitigating Bias in Clinical Encounters.” All JMP faculty who teach this content regularly participate in faculty retreats with ongoing training in antiracism. Detailed facilitator guides were developed for each session to guide faculty instructors [see an excerpt from the Facilitator Guide for Mitigating Bias in Clinical Encounters, Part 2 - Additional File 1].

### Setting and population

This study took place at the UC Berkeley-UCSF JMP, a five-year graduate/medical degree program with a vision of advancing health equity and social justice. Inclusion criteria included medical students at the JMP who were undergoing their pre-clerkship training during the 2022–2023 academic year (AY). Exclusion criteria included medical students at the JMP who had begun a clinical clerkship rotation during the 2022–2023 AY. All medical students who were invited to participate were given the option to opt-out of the study. Each cohort is comprised of 16 medical students who spend their pre-clerkship years at UC Berkeley engaging in a curriculum centered around student-led inquiry.

### Study design

We used a sequential, mixed methods approach using pre/post surveys and semi-structured individual interviews in our study to measure students’ reaction and learning after completing the ARC and elicit their feedback on ways the curriculum could be improved for future iterations. Using the antiracism learning objectives developed by core faculty at the JMP as a guide (Table 1), the study team developed a pre/post survey using a standard 5-point Likert scale to assess knowledge of these learning objectives before and after implementation of the ARC, comfort level in talking about race/racism, familiarity with identifying tools for patient-centered communication (PCC), and familiarity with identifying impact of provider bias on medical decision making (MDM).

**Table 1:** Antiracism curriculum learning objectives

Recognize and unpack your own assumptions and biases about patients (implicit and explicit)
Recognize your privilege in a given setting, and proactively work to address consequent power differentials
Understand and recognize the ways in which patients experience racism and trauma in medical settings
Understand and explore the history of racism in medicine
Recognize race-based medicine at practice
Understand how structural racism informs disparities and the patient experience
Develop skills for interprofessional collaborative care
Deconstruct dominant narratives and recognize language bias in verbal/written communication
Develop effective skills for structural and system change
Develop excellent clinical skills that you can apply to ALL patient scenarios

The qualifiers of the Likert Scale were as follows: (1) Not at all, (2) A little, (3) Somewhat, (4) Quite a bit, and (5) Very. The survey also collected demographic data (i.e. gender identity, racial/ethnic identity, and sexual orientation) for students who opted into the study. Once students gave consent to participate, they created a unique Study ID so that their pre/post survey could be paired upon completion of the study for data analysis. The study team constructed an interview guide (see supplementary material S1) using principles from the “Equity-Minded Inquiry Series” at the USC Rossier School of Education [15]. In this guide, authors provided step-by-step instructions on how to develop and conduct student interviews focused on racial equity.

The study was deemed exempt by the UCSF Institutional Review Board (IRB# 22-37293). This work was conducted according to the ethical principles outlined in the Declaration of Helsinki [16]. All participants gave their informed consent to participate in the study. Prior to the study, we piloted and vetted the survey and interview guide questions with UCSF medical students during the “Health Equity Scholarship in Progress” Forum available at UCSF.

### Data collection

Our ARC was implemented among a cohort of 32 pre-clerkship medical students over the 2022–2023 academic year. The project was introduced to the students at the beginning of their academic year during which they were asked to voluntarily participate. Surveys were administered in Qualtrics (Qualtrics, Provo, Utah) and JMP faculty provided time during class for students to fill out the pre/post surveys. At the end of the academic year in May 2023, email invitations were sent out to the students asking for volunteers to participate in the individual interviews. Students were informed ahead of time that author R.J. did not participate in developing the curriculum to encourage honest conversation. Additionally, they were assured that their responses would remain anonymous when shared with those who were involved in developing the curriculum. Author R.J. conducted all the interviews in a private area over Zoom (Zoom Video Communications, San Jose, California). The interviews were audio recorded and then professionally transcribed using Descript (Descript, San Francisco, California). Interviews continued until thematic saturation was reached.

### Data analysis

The study team went through the pre/post data collected on Qualtrics and matched responses using the unique Study IDs created by the students. Authors then performed Wilcoxon signed-rank test analysis on the paired pre/post data to assess if learners’ knowledge of the antiracism learning objectives was significantly different after completing the ARC. For this study, statistical significance was defined as a threshold of *p* < 0.05. Various other descriptive statistics were also performed on the paired surveys to summarize and organize the data.

After the interviews were transcribed using Descript, the transcripts were uploaded into the Dedoose (Manhattan Beach, California) coding software. Authors utilized framework analysis and open coding to identify themes in the qualitative data from the transcripts [17]. Two authors (R.J. and O.E.) independently coded the first 3 de-identified transcripts and once there was agreement through iterative discussion between the two investigators, they developed a code book that was used as a reference for the remaining transcripts. Author R.J. subsequently coded all remaining transcripts and examined themes to understand how the students perceived the ARC and elicit feedback on what could be improved for subsequent years.

## Results

Overall, 28/32 (87.5%) pre-clerkship medical students at the JMP consented to participate in the study, with 20/32 (62.5%) completing both the pre/post surveys. Of those who consented to participate, 13/28 (46.4%) agreed to complete an individual interview. Demographic data showed that 27% of survey respondents and 23% of interview participants come from underrepresented minority (URM) groups, as defined by the UCSF Office of Diversity and Outreach (Table 2).

**Table 2:** Demographic Data of Student Participants

**Joint Medical Program Year**	**Surveys (*N*** **= 28)**	**Interviews (*N*** **= 13)**
JMP 1	16	12
JMP 2	12	1
**Gender Identity**	**Surveys**	**Interviews**
Cis-female	58%	62%
Cis-male	29%	31%
Other	*	*
Prefer not to respond	*	*
**Racial/Ethnic Identity**	**Surveys**	**Interviews**
Caucasian or White	23%	32%
Asian (Including South Asian, Southeast Asian, and Asian American)	47%	38%
Hispanic or Latino/Latinx	17%	*
Middle Eastern	*	*
North African	*	*
Prefer not to respond	*	*

* All counts < / = 2 suppressed for participant anonymity

**Fig. 1:**
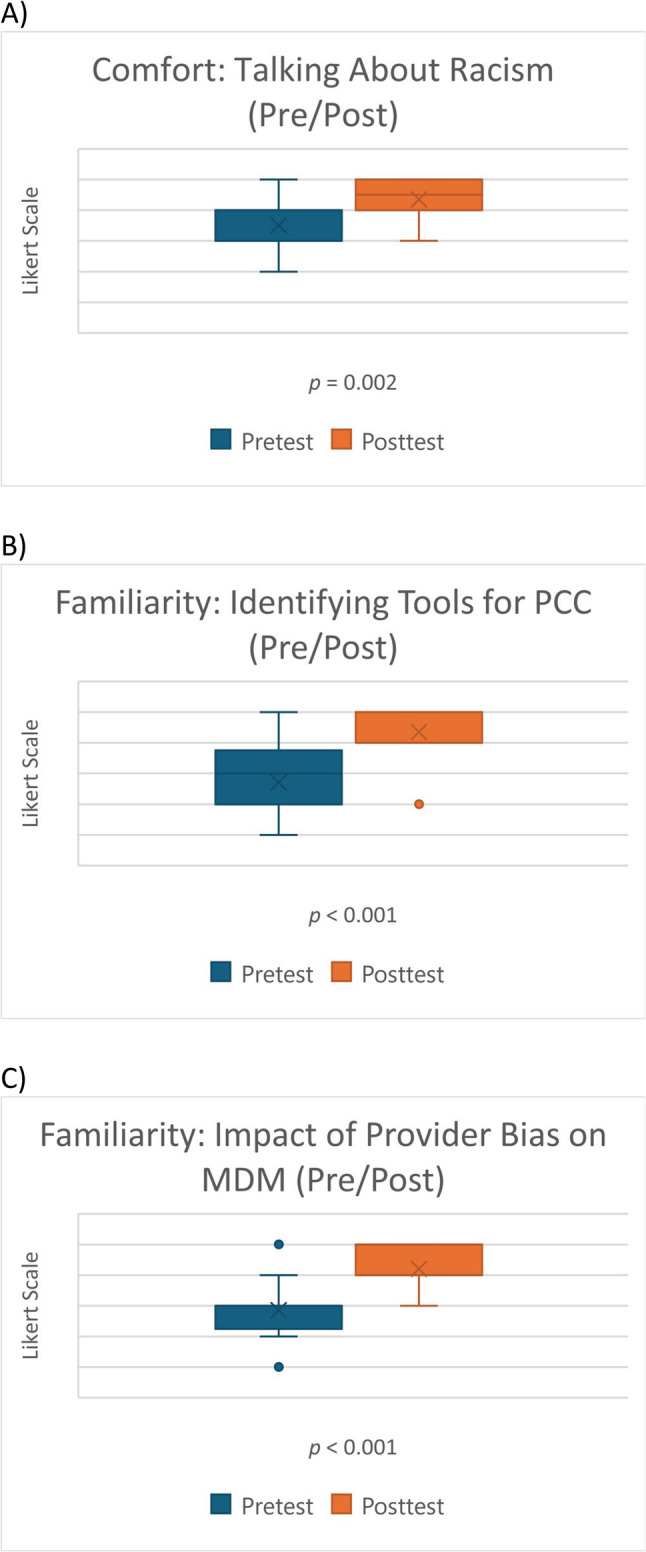
Pre/Post Box Plots of Students’ Comfort Level and Familiarity with Antiracism Topics with Wilcoxon Signed Rank Test Analysis. (**A)** Racism, (**B**) Identifying Tools for PCC, and (**C**) Impact of Provider Bias on MDM. *PCC = Patient-Centered Communication, MDM = Medical Decision-Making

In total, students in this study received 108 total hours of clinical skills training, within which we embedded ARC-associated training and education. Not surprisingly, the pre- and post-data collected to assess familiarity with the ARC learning objectives reflects the benefit of having dedicated time to discuss these topics. Prior to the ARC, 44% of students reported being “quite a bit” or “very comfortable” talking about racism, 17% said they were “quite a bit” or “very comfortable” identifying tools for patient-centered communication (PCC), and 17% said they were “quite a bit” or “very comfortable” identifying the impact of provider bias on medical decision making (MDM). After engaging with the ARC, 88% of students reported being “quite a bit” or “very comfortable” talking about racism, 94% said they were “quite a bit” or “very comfortable” identifying tools for PCC, and 88% said they were “quite a bit” or “very comfortable” identifying the impact of provider bias on MDM. Students’ familiarity with each of these topics after completing the ARC reached statistical significance (Fig. 1A-C).

When students were asked to rate their knowledge of how historical context contributed to existing healthcare disparities on a Likert Scale from 1 to 5, the mean prior to participating in the ARC was 2.85 and after participating in the ARC the mean was 4.25. Students’ knowledge of antiracism topics reached statistical significance for 13/14 (93%) of the learning objectives outlined for the ARC (Table 3).

**Table 3:** Pre/Post Wilcoxon Signed Rank Test Analysis of Antiracism Curriculum Learning Objectives

Learning Objective	Mean Likert Score (Pre)	Mean Likert Score (Post)	Median Likert Score (Pre)	Median Likert Score (Post)	*p*-Value*	Effect Size*
Antiracism inquiry	2.8	4	3	4	*p* < 0.001	0.8
Power imbalances in medicine	3.7	4.45	4	5	*p* < 0.01	0.7
Structural competency	2.3	3.9	2	4	*p* < 0.001	0.9
Race-based medicine	2.9	4.55	3	5	*p* < 0.001	0.9
Levels of racism	3.4	4.55	3	5	*p* < 0.001	0.8
Stigmatizing language	3.65	4.5	4	5	*p* = 0.019	0.7
Dominant narratives	3.1	4.2	3	4	*p* < 0.001	0.9
Implicit bias	4	4.5	4	5	*p* = 0.009	0.8
Medical myths	2.8	4.35	3	4	*p* < 0.001	0.8
Race corrections	2.85	4.45	3	5	*p* < 0.001	0.8
Theory of weathering	2.6	4.05	3	4	*p* < 0.001	0.8
Health disparities experienced by diverse racial and ethnic groups	4.2	4.55	4	5	*p* = 0.059	0.6
Recognizing the ways in which patients experience racism in medical settings	3.55	4.5	4	5	*p* < 0.001	0.9
Understanding the history of racism in medicine	2.85	4.25	3	4	*p* < 0.001	0.8

Thematic analysis of the transcripts identified 5 themes related to students’ engagement with the ARC: (1) effective instructional activities for teaching ARC, (2) students’ overall impression of ARC content, (3) ways to improve the ARC, (4) impact of students’ identity on their ability to engage with ARC, and (5) students’ prior experience with antiracism topics before ARC (Table 4).

**Table 4:** Thematic Analysis and Key Quotes from Interview Transcripts

Theme	Key Quotes
Effective Instructional Activities for Teaching ARC	“I feel like the day that we had the interpreters, was … probably the best like clinical skills session so far. Cause it just … opened up so much more like conversations about … access and how … stigma is provided on … the provider end and how that gets … written in a chart. And I feel having an opportunity like this with the interpreters just gave a lot more room for … conversations that weren’t always built into … some of the other sessions.” “Like how to describe a patient in crafting your history to be more mindful of like deconstructing dominant narratives and like ensuring that the way that we talk to and about patients in clinical care, like does not reinforce like structural and interpersonal racism. But that one felt like so well situated for like our clinical skills training where like racism can so easily come in.”
Overall Impression of ARC	“ I think it’s just fundamental of any medical school curriculum. And I wish more schools would have this sort of component in their education. The conversations we have are purposeful and very impactful” “I think just that the time constraint is a really big factor for me. For me personally, I feel like I do learn so much and I just wish that that was happening more often because I leave clinical skills feeling like I learned something so new and like I’m really excited about it, but then it just feels so rushed that I’m still don’t really know why I am doing this.”
Ways to Improve the ARC	“I feel like after reading about disparities in literally every field, it gets a little bit like demotivating and not seeing how people are actually addressing this or making change. Perhaps introducing us to programs or organizations that are actively doing something about it. It’s not just research paper, you know? And when you read that, you can’t just imagine what are people doing? How can I do it? “I think it wasn’t originally built into our system. It’s not built into our medical education. I think it is something that still needs to be integrated and have everyone who’s teaching understand why it’s important. Have everyone who is going into medical education, understand why it’s important.”
Impact of Students’ Identity on Ability to Engage with ARC	“I think my growth personally has been from learning the perspectives of other people and seeing how that that’s pretty different from the perspective that I grew up with and just being able to learn that more deeply” “I hold privilege of identifying as a white person and aware of how that shifts dynamics in clinical encounters where when I’m working with people of color or like classmates of color. Am I overtaking people? Am I allowing space for others to speak up? How much space am I taking up?
Students’ Prior Experience with Antiracism Topics prior to the ARC	“I didn’t know how much was wrong with the medical system, especially regarding racism and learning about race-based medicine, like GFR. Yeah. It was definitely something like a wake-up call for me and then being like, how can I be better for my patients” “I feel like a lot of it was self-education and discussions with peers on my own time type of thing”

The most effective teaching activities included a simulated clinical patient encounter that allowed students to interact with medical interpreters and standardized patients and a clinical reasoning session where students deconstructed dominant narratives in medicine. Students commonly described the ARC as “impactful” but also noted that there was “not enough time” dedicated to it. Additionally, most students interviewed wanted even more integration of the ARC with other parts of their medical education curriculum and expressed the desire for ARC content to shift away from “raising awareness about health inequities” to a more “solution-oriented” focus. A key idea that many students brought up, often unprompted, was a worry of how this education would translate outside of the JMP space. There was a recognition that not every medical student receives education on antiracism and many healthcare institutions don’t have leaders dedicated to antiracism efforts. Students’ personal identities impacted the way they engaged with the ARC in several different ways. While there was no expectation for students to share any of their lived experiences, many were excited to share their unique perspectives that were shaped by the way they grew up and interacted with the world, while equally looking forward to the opportunity to learn from their peers. This was with the caveat that a proportion of students who came from marginalized backgrounds acknowledged that sharing their perspective so peers could learn from their lived experiences was harmful and retraumatizing to relive traumatic experiences of oppression. Additionally, those students who identified as “White” or “Caucasian” expressed fears of “taking up too much space” or “saying the wrong thing and committing a microaggression.” Finally, many students reported they had no prior experience with antiracism topics before the ARC, and of those who did, the most common exposures were through personal experiences. There was great appreciation for the ARC providing historical background for existing healthcare inequities.

## Discussion

There is a clear need for a shift in the design of medical education so that trainees are better equipped to not only recognize racial and ethnic health disparities but also develop tangible skills to address these inequities both in clinical practice and by impacting systemic change. Despite the JMP’s mission of advancing health equity and social justice, the students in this study still had low initial scores on the knowledge assessments, highlighting the current need for antiracism education. It is clear from our study that students found the most value in those sessions that focused on efforts to actively build skills in alignment with antiracism principles (i.e. practice working with medical interpreters, mitigating bias in clinical encounters, etc.). Yet, these skill-building sessions only comprised < 25% of the whole ARC curriculum. They also highlighted the point that just reading about disparities is not enough; there is a desire to learn how this recognition of inequity can be translated to meaningful change within our healthcare system. Historically, antiracism teaching has been ancillary and separated from clinical context. This spiraling curriculum integrated antiracism and clinical skills learning objectives longitudinally over two academic years. In general, the curriculum was well-received by learners and achieved the objective of improving the ability of pre-clerkship medical students to understand various antiracism topics in medicine and begin to identify tools for PCC and the impact of bias on MDM. It also aligned with the principles of student-led inquiry given the JMP’s mission of advancing health equity and social justice. Prior studies indicate that faculty discomfort with antiracism material and lack of formal faculty development materials to prepare educators on how to effectively teach about antiracism are two of the most common reasons students report when asked about barriers to successfully incorporating antiracism within medical education [18]. This discomfort and unpreparedness lead to a superficial engagement with antiracism topics that is detrimental to students’ learning as it often does nothing more than acknowledge that racism exists in medicine without any skill-building on how physicians can address and counteract it [19]. Expertise amongst medical educators taking on the responsibility of teaching antiracism is necessary to ensure proper time and discussion is given to these important topics. Finding time for this within the pre-clinical medical education curriculum will continue to be a crucial issue to address, as a core part of the clinical skills curriculum is teaching trainees to be excellent clinicians, which is a key component of delivering antiracist care. Equally important is recognition that there will be different levels of learners with unique perspectives in each classroom setting, highlighting the need for educational strategies such as affinity groups (Lewis, [14]) as one strategy to further enhance antiracism discussion in medical education.

Our study had a small sample size and was conducted at a single institution, limiting the generalizability. Furthermore, it sampled trainees who sought out a medical education program focused on health equity and social justice and thus is not representative of all medical trainees. Additionally, the design of the study did not allow for an in-depth analysis of the skill-building component of the ARC and instead was an overall evaluation of the students’ impression of the curriculum and knowledge gained, levels 1 and 2 of the Kirkpatrick Model. Despite this, our mixed-methods methodology allowed us to objectively obtain feedback on what parts of the curriculum worked well and what needed to be improved for the future. It also highlighted the lack of exposure to antiracism topics in pre-medical education, as many traineesreceive no teaching on this prior to starting at the JMP. Future directions for this study include piloting the ARC with a larger sample of medical students not in the JMP and surveying clerkship medical students who have gone through the ARC to see if they were able to apply any of the skills they learned in clinical practice. Even though the average medical school class size (~ 100 medical students per year) is significantly larger than the class size at the JMP, the ARC sessions can be adapted and integrated into existing clinical skills curricula at larger institutions as many of them already have built-in time for students to engage with standardized patients and discuss best practices for clinical skills. Perhaps most importantly, the feedback provided by the students will be used to create a subsequent iteration of the ARC that places more emphasis on sessions that develop tangible, anti-oppressive skills and highlights a solution-oriented approach to tackling disparities within healthcare.

## Conclusions

A key finding from this study is that while many medical students are aware of health disparities, few understand the history behind why these disparities exist and how they are perpetuated in current clinical practice. This study demonstrates that it is feasible to integrate a longitudinal antiracism curriculum into the existing clinical skills block during the pre-clinical years of medical school education. Furthermore, this type of curriculum was well-received by the students who participated in the study and increased their knowledge on core antiracism topics. However, while the ARC begins to develop a new model for how to shift the traditional discourse around antiracism principles to one that emphasizes skill-building and evidence-based solutions, further refinement of the curriculum is necessary. It also needs to be extended into the clinical education space to promote lasting behavioral changes. Recognition of current gaps in care and an active effort to integrate antiracism practice into formal medical education are crucial steps towards implementation of potential solutions to mitigate health inequities so that we can begin to enact real change and improve outcomes for all our patients.

## Supplementary Information

Supplementary Material 1.

Supplementary Material 2.

## Data Availability

All data generated or analyzed during this study are included in this published article.

## Abbreviations

DEI: Diversity, equity, and inclusion
AAMC: The Association of American Medical Colleges
ARC: Antiracism curriculum
JMP: Joint Medical Program
PCC: Patient-centered communication
MDM: Medical decision making
