# Supplementary material for: Assessing the effectiveness of an antiracism clinical skills curriculum for medical students: a single institution mixed methods study
Source: BMC Med Educ. 2026 Feb 21;26:511. doi: 10.1186/s12909-026-08854-z (PMC13032428; doi:10.1186/s12909-026-08854-z)
Supplement: Supplementary file 1 — Supplementary Material 1. [file 12909_2026_8854_MOESM1_ESM.docx]

**S1. Interview Guide**

Sample Transcript of Semi-Structured Questions:
The sub-questions listed underneath Q1-11 may be asked to stimulate responses from the interviewees. Question 7 (highlighted and bolded in **blue**) will only be asked to the JMP2 students.

*Opening and Rapport Building*

1. Can you tell us a little bit about yourself?
   1. How do you identify your race and ethnicity?
   2. How do you identify your gender and what pronouns should we use when referring to you?
2. Why did you choose to be a student in the JMP?
   1. What made you say yes to participating in this study?

*Characterization of Instructors and Experiences in the Classroom*

1. What are three words that you would use to describe your experiences with the antiracism component of the Clinical Skills curriculum?
   1. For each word selected, please share why you chose that descriptor.
2. If you think about the things like the syllabus for the course and the kinds of activities that you did in class, is there a specific activity, reading, or interaction that stands out for you?
   1. How did that activity make you feel or what did it make you think?
3. How well do you think your instructors do with giving all students the opportunity to ask/3 and answer questions?
4. In what ways do you think your identity affects your experience in this course?
5. **Now I am going to transition to questions about specific anti-racism focused sessions within the Clinical Skills curriculum:**
   1. **On the session on Mitigating Bias Part 1 (8/30/22), where it focused on patient ancestry, social history, and deconstructing dominant narratives, what would be two words that you would use to describe that experience and why?**
      1. **What components of the session were most impactful and why?**
      2. **What components of the session would you change and why?**
      3. **What aspects (if any) of the session will you carry forward to your clerkships and future clinical encounters?**
   2. **On the session on Mitigating Bias Part 2 (1/17/23), where it focused on using tools to evaluate research on race/ethnicity and applying anti-racist skills to a clinical case, what would be two words that you would use to describe that experience and why?**
      1. **What components of the session were most impactful and why?**
      2. **What components of the session would you change and why?**
      3. **What aspects (if any) of the session will you carry forward to your clerkships and future clinical encounters?**
   3. **On the sessions on trauma-informed care that you have received thus far, what would be two words that you would use to describe that experience and why?**
      1. **What components of the session were most impactful and why?**
      2. **What components of the session would you change and why?**
      3. **What aspects (if any) of the session will you carry forward to your clerkships and future clinical encounters?**

*Knowledge/Training*

1. Prior to medical school, how much training or education did you get about structural racism?
   1. Where did you get this training or education from?
   2. Why is it important to learn about structural racism?

*Skills/Encounters*

1. How comfortable do you feel taking care of patients from culturally diverse backgrounds?
   1. What are the challenges or barriers you anticipate facing?
   2. How do you think your own personal biases affect your interactions with patients and their families?

*Evaluation*

1. Overall, how would you rate the antiracism curriculum implemented this year?
   1. What are some potential challenges you see to integrating training about anti-racism praxis into medical education?
   2. What advice would you give to instructors who are developing and teaching this course?
      1. How can instructors best support students who share diverse identities?
   3. What are ways the curriculum could be improved?

*Closing*

1. Is there anything that I did not ask about your instructors or experiences with the course that you would like to share?
