# Supplementary material for: Assessing the effectiveness of an antiracism clinical skills curriculum for medical students: a single institution mixed methods study
Source: BMC Med Educ. 2026 Feb 21;26:511. doi: 10.1186/s12909-026-08854-z (PMC13032428; doi:10.1186/s12909-026-08854-z)
Supplement: Supplementary file 2 — Supplementary Material 2. [file 12909_2026_8854_MOESM2_ESM.pdf]

JMP Clinical Skills Curriculum

Mitigating Bias in Clinical Encounters: Part II

Facilitator's Guide

Session Overview:

During this session:

1. *Students will discuss and critique a research article relating to race and glaucoma making use of a new standard for publishing on racial health inequities.*
2. *Students will work through a clinical case that has been impacted by implicit bias, integrating anti-racist approaches with clinical reasoning to mitigate these impacts.*

Session Objectives:

By the end of this session, learners will:

- Enumerate the steps in appraising the use of race in clinical research from an anti-racist perspective
- Appraise the validity of an article from the primary literature with regards to its use of race
- Recognize and address errors from implicit bias and cognitive biases in medical decision making.
- Appreciate the role of humble inquiry.

Session Timeline:

Time		Topic
9:10 – 9:20	10 min	Session Overview and Intro of Faculty
9:20 – 10:35	75 min	Exercise: Critical Appraisal of Research with Anti-Racist / Health Equity Lens (**Group A**)<br><br>Exercise: Utilizing Clinical Reasoning and Bias Mitigation to Address Diagnostic Error (**Group B**)
10:35 – 10:45	**10 min**	**BREAK**
10:45 – 12:00	75 min	Exercise: Utilizing Clinical Reasoning and Bias Mitigation to Address Diagnostic Error (**Group A**)<br><br>Exercise: Critical Appraisal of Research with Anti-Racist / Health Equity Lens (**Group B**)

Pre-Session Preparation:

Clinical Mentors

- Read this facilitator guide.
- Higginbotham et al. The Ocular Hypertension Treatment Study: Topical Medical Delays or Prevents Primary Open-angle Glaucoma in African American Individuals. Arch Ophthalmol 2004; 122:813-820.
- Boyd et al. On Racism: A New Standard For Publishing On Racial Health Inequities. Health Affairs 2020.
- Review the Critical Appraisal of Race in Medical Literature (CARMeL) Tool
- Listen to the EPISODE 2 - THE DEI SHIFT Podcast : “Seize the DEI: Shaping the Future of I.M.” - Minute 19-35 (Dr. Denise Connor’s Portion)

Required Preparation for Students

- Higginbotham et al. The Ocular Hypertension Treatment Study: Topical Medical Delays or Prevents Primary Open-angle Glaucoma in African American Individuals. Arch Ophthalmol 2004; 122:813-820. (*focus on abstract, introduction, methods, and “comment” – i.e. discussion)* - ~30 minutes
- Boyd et al. On Racism: A New Standard For Publishing On Racial Health Inequities. Health Affairs 2020. - *~25 minutes*
- Review the Critical Appraisal of Race in Medical Literature (CARMeL) Tool **-** ~15 minutes
- Listen to the EPISODE 2 - THE DEI SHIFT Podcast : “Seize the DEI: Shaping the Future of I.M.” - Minute 19-35 (Dr. Denise Connor’s Portion)

Optional Pre-Session Reading/Preparation:

- Flanagin A, et al. Updated Guidance and the Reporting of Race and Ethnicity in Medicine and Science Journals. JAMA. 2021; 326(7):621-627.
- Halawa OA, et al. Racial and Socioeconomic Differences in Eye Care Utilization Among Medicare Beneficiaries with Glaucoma. Ophthalmology. 2022; 129(4): 397-405.

- # Detailed Session Facilitator Guide:

Intro and Check-in (10 minutes): 9:10-9:20

- Review session overview, objectives, and schedule
- Check-in regarding any questions about the pre-session materials.

Exercise: Critical Appraisal of Research – (LG – 75 minutes): 9:20-10:35

1.) Have the students **summarize the findings** of the **Higginbotham et al OHTS study**, which describes outcomes due to glaucoma among African American individuals with elevated intraocular pressure who receive either topical medication or observation alone, comparing these outcomes with individuals who did not identify as African American. *(10 minutes)*

- **Key findings**:
  - *Study overview:* Individuals with **elevated intraocular pressure but good visual acuity, normal visual fields, and normal optic discs** were **randomized to receive topical ocular hypotensive medication or undergo observation** and were then **followed for outcomes due to primary open angle glaucoma** (visual field abnormalities or optic disc deterioration). The first randomized trial on the prevention of primary open angle glaucoma to enroll many African American participants, who have a high prevalence of glaucoma and glaucoma-related blindness. *[Consider commenting here or elsewhere on why the authors chose AA instead of Black and how that shapes or may impact folks' thinking and observations (including whether all participants truly were AA or if this is language that was being used loosely)]*
  - *Treatment outcomes by race:*
    - Among **African American** participants**,** the percentage developing **primary open angle glaucoma (POAG) in the medication group (8.4%) was significantly lower than in the observation group (16.1%)** (HR 0.50, 95% CI 0.28-0.90).
    - Among **other (not self-identified as African American)** participants, the percentage developing **POAG in the medication group (4.4%) was significantly lower than in the observation group (11.6%)** (HR 0.36, 95% CI 0.23-0.57).
    - Though a **trend is apparent (HR 0.50 vs. 0.36) the protective effect of medication** among African American participants was **not statistically different** from that among other participants.
  - *Race as predictor of glaucoma*:
    - In **univariate analysis**, self-identified **African American race was associated with a 71% increase in risk of developing POAG** compared to other participants (HR 1.71, 95% CI 1.2-2.4), **twice the hazard in the medication group and 58% higher hazard in the observation group**, despite having the similar baseline and follow-up IOPs.
    - After **multivariate adjustment**, including as vertical/horizontal **cup-disc ratios (larger at baseline in African American participants), central corneal thickness (thinner at baseline in African American participants)**, age, sex, history of diabetes, systemic hypertension and heart disease, intraocular pressure, and pattern standard deviation, **African American race was no longer statistically significantly associated with an increased risk of developing POAG** (HR 1.1, 95% CI 0.76-1.66). Of note, the study did not differentiate sex and gender.

Now we will examine this article and some of the broader body of literature, from the framework described in "On Racism: A New Standard for Publishing on Racial Health Inequities." (*40 min - #2-4*)

2.) Boyd et al, calls on researchers **to define race, to do so from a sociopolitical framework rather than a biological one, and specify the reason for its use**. They caution authors **never to offer genetic interpretations of race because such suppositions are not grounded in science**, and if race and genetics are expressed jointly, to carefully delineate the intended implication. How have the authors of this article defined race? How have they described the justification for inclusion of the use of race?

- The authors do explicitly define the race variable as <u>self-identified</u> African American (not of Hispanic origin) or "other" (including white, Hispanic, Asian, American Indian or Alaskan Native, and unknown), though they do not otherwise define race itself.
- The study design hypothesizes that one race would respond differently to a pharmaceutical agent, which implies a certain degree of genetic interpretation.
- The authors provide the background that glaucoma is the leading cause of blindness among African American individuals with 4-5 times the prevalence of primary open angle glaucoma as white individuals. The original OHTS article only showed a trend towards a protective effect of treatment among African Americans. The aim of this study was to examine whether topical medication would in fact prove beneficial among African American individuals with a longer period of follow-up.
- <u>*PROBE: What assumptions might the authors be making in relation to considering race a biological or genetic trait? How can this subtly underpin a research question without being stated outright?*</u>

3.) One of the take-home points of the study discussed by the authors is that the increased risk of primary open angle glaucoma among participants who self-identified as African American, is attributable to specific clinical factors rather than race itself. In the *comment* section (page 819, 2nd paragraph of the 2nd column), the authors further discuss that race as a construct of classification and the self-identification of race are problematic. **Discuss ways in which this framing is helpful and ways in which it may not provide the whole picture?** (PROBE: (1) Sociopolitical vs. biological framing of race. (2) Unmeasured confounding – i.e. how can racism be considered if race is not a significant predictor after multivariate adjustment?)

- <u>*PROBE: What did Boyd say about race and genetics? (The Boyd article states emphatically "Never offer genetic interpretations of race because such suppositions aren't grounded in science.")*</u>

4.) In the *comment* section (page 819, beginning with the last sentence of the 1st column), the authors of the OHTS study provide a list of factors that may contribute to increased prevalence of glaucoma and glaucoma-related blindness in individuals of African origin and then go on to discuss how their findings may or may not address some of these explanations. They do not, however, explicitly name racism (whether interpersonal, institutional, or internalized) and the mechanism by

which it may operate. **How might the issues they describe tie into a substantive discussion of racism, including structural racism?** (PROBE: **How might they think about next steps in research, policy, or clinical practice with racism in mind? → Could include use of "experiences of racism" scale to more closely evaluate variables of interest.**)

- *Consider talking about the potential harms for study participants having to remember experiences of racism and how that could be mitigated if planning to study that topic – e.g., how can research itself be trauma informed.*
- *Think about the structural barriers for publishing articles on racism, power dynamic of editors and reviewers and their lenses.*

5.) Students will now apply the **CARMeL Tool (Appendix A)** to appraise this article. Divide the students into three groups. Assign each group to ONE of the appraisal domains (internal validity, external validity, or applicability). Each group will answer the questions from the tool that are included in their assigned domain. Let them know to select a representative from the group who will report back to the larger group after the breakout. Allow approximately *15 minutes* for the break-out groups. After 15 minutes and/or all groups are done, bring them back into one large group and ask each group to report back their findings. (*25 minutes total*)

- Detailed Study Overview (if needed for discussion):
  - *Study Design:* Randomized controlled trial
  - *Inclusion Criteria*: age 40-80; IOP 24-32 mmHg in one eye, 21-32 mmHg in fellow eye; with normal visual fields; and normal optic discs
  - *Exclusion Criteria*: visual acuity worse than 20/40 in either eye, previous intraocular surgery other than uncomplicated cataract, diabetic retinopathy or other diseases that cause visual field loss or optic disc abnormalities
  - *Sample:* 1636 individuals randomized; 203 participants who self-identified as African American randomized to receive topical medication and 205 participants who self-identified as African American randomized to the observation group
  - *Intervention:* Topical ocular hypotensive medication (all commercially available) to achieve target IOP reduction
  - *Follow-up:* every 6 months
  - *Primary Outcome*: development of primary open angle glaucoma in one or both eyes, defined as reproducible visual field abnormalities or a clinically significant reproducible optic disc deterioration attributed to POAG by the masked endpoint committee

BREAK – 10 minutes: 10:35-10:45

<u>Exercise: Diagnostic Error - Clinical Reasoning + Mitigating Bias – (LG – 75 minutes)</u>: 10:45-12:00

You are going to read a clinical encounter where you are likely to see problems embedded in the way they interface with the patient*. You are a co-resident in the pediatric emergency department when a pediatric resident presents the following H&P regarding a patient presenting with epistaxis** (Pairs*** *- 5 minutes*)**:**

Resident presentation: *This is a 17-year-old boy (he/him) with history of allergic rhinitis, but otherwise previously healthy, who recently immigrated from Guatemala, and presents with chronic nose bleeds. I obtained the history with a video Spanish interpreter. The bleeding first started about 3 months ago and was occurring about 3 times per month. In the past three to four days, the nosebleeds have become more frequent. He also endorses some chronic nasal congestion. He reports no trauma to his nose, fever, easy bruising or other sources of bleeding, and there is no family history of bleeding disorders.*

He was seen at an outside hospital emergency department yesterday and was advised to initiate the nasal steroid that he had been prescribed previously and Vaseline. He is here today because he did not feel that they answered his questions and he is still having nose bleeds. For some reason, he seems very worried about the bleeding and is perseverating on how much blood that seems to be coming out. I wonder if he might be developmentally delayed. His HEADSSS assessment is negative; he reports no substance use and feels safe at home.

On exam, his vital signs are normal without tachycardia. He has dried blood in bilateral nares, and his nasal mucosa is somewhat erythematous. There is no active bleeding. Otherwise, his examination is normal without lymphadenopathy, hepatosplenomegaly, rash, ecchymoses, or petechiae.

After the resident provides their assessment and plan, you incorporate a bit of feedback and teaching:
Nice job obtaining a complete history and providing a clear sense of timeline and progression over time. You also did well evaluating for systemic causes of bleeding. I also am glad that you made use of an interpreter and performed a HEADDSS assessment. Most epistaxis comes from the Kiesselbach plexus of veins in the anterior nose. However, for any patient presenting with epistaxis, I evaluate for the following symptoms that raise concern for posterior bleeding:

- *Bilateral bleeding (also might suggest bleeding diathesis)*
- *Sensation of bleeding in back of throat, swallowed blood, hematemesis, melena*
- *Prolonged bleeding despite appropriate application of anterior pressure*
- *Furthermore, prolonged or recurrent bleeding (whether anterior or posterior) or other mucosal bleeding can be signs of a bleeding disorder or other systemic cause.*

The resident adds:
When the bleeding occurs, he tends to just wait for the bleeding to stop, and it does so after about 10-15 minutes. He has tried to stop the bleeding by plugging his nose, but I don't believe he is actually

squeezing his nose appropriately at the tip. I'm not sure which side the bleeding comes from generally, though it did seem bilateral on exam.

Additional Epistaxis Notes for Facilitator:

Nosebleeds in children have a variety of etiologies, ranging from self-limited mucosal irritation to life-threatening neoplasms. Epistaxis is rare in children younger than two years (approximately 1 per 10,000) and should prompt consideration of trauma (intentional or unintentional, including asphyxiation) or serious illness (e.g., thrombocytopenia). Overall, epistaxis that occurs in children younger than 10 years usually is mild and originates in the anterior nose, whereas epistaxis that occurs in individuals older than 50 years is more likely to be severe and to originate posteriorly. Common causes of nosebleeds in children include **mucosal dryness**, trauma (most commonly **nose-picking**), foreign body, and rhinitis (allergic, infectious, or related to mucosal irritation). Less common, but important causes of nosebleeds to remember include bleeding disorders and other **systemic diseases (e.g. ITP, VWD, hypertension)**, **tumors (e.g. juvenile nasopharyngeal angiofibroma in adolescent boys, rhabdomyosarcoma)** and post-traumatic pseudoaneurysm of the internal carotid artery or carotid-cavernous sinus fistulae.

Anterior nosebleeds, usually arising from Kiesselbach's plexus, are most common (accounting for approximately 90 percent of nosebleeds in children) and are almost always self-limited. Anterior nosebleeds usually result from mucosal dryness, trauma, or irritation, although many cases are idiopathic. Posterior nose bleeds are unusual in children and usually due to significant nasal trauma. Posterior bleeds usually arise from the posterolateral branches of the sphenopalatine artery. One important cause of posterior bleeding that is important to know, and is the diagnosis in this case, is **juvenile nasopharyngeal angiofibroma** (seen exclusively in adolescent males and commonly associated with nasal congestion, facial fullness, or even facial swelling). *The prevalence in males may be explained by high androgen receptor (AR) expression suggesting that JNA is androgen dependent. - Schick B, Rippel C, Brunner C, Jung V, Plinkert PK, Urbschat S. Numerical sex chromosome aberrations in juvenile angiofibromas: Genetic evidence for an androgen-dependent tumor? Oncol Rep. 2003;10:1251–5.*

Clinical Pause (*35 minutes***)**
(*#1/#2 - Pairs + Group Share in LG →* *20 minutes;* ***#3 - LG or Pairs + Group Share in LG →*** *15 minutes***):**

1.) Write an initial problem representation.

Many reasonable options, one example:

17-year-old boy (he/him) with history of allergic rhinitis, not currently on treatment, with associated nasal congestion presenting with acute on chronic, worsening epistaxis that may be bilateral.

2.) You may have noticed some aspects of the presentation that seem problematic. What did you notice? Highlight and discuss aspects of the presented H&P that seem potentially vulnerable to the introduction of bias into clinical reasoning and that you would like to pay attention to as you prepare for your own clinical interaction with the patient.

- *Patient is non-English speaking and a recent immigrant, which may increase the risk that providers have not taken the time to understand his perspective and that he may not be well-connected with medical resources such as primary care.*
- *The resident evaluated the patient to potentially have intellectual disability. Patients with intellectual disability and other disabilities are affected disproportionately by bias. It is also possible that the patient does not have intellectual disability, and that this conclusion derived from disconnect in communication and understanding. Of note, intellectual disability is usually diagnosed through longitudinal relationships and in-depth assessments rather than a single emergency department visit. The resident also used the term 'developmental delay' which is not specific.*
- *There is discussion of medication non-adherence as well as more than one ED visit; from the perspective of the medical establishment, this may represent a patient who is both overly concerned and is not following medical advice. Adherence is often used as a thinly veiled disguise for discounting patients as problematic, rather than to take the time to respectfully explore why a patient is not taking a medication. In this case, the medication is not working for him! But access, education, health literacy, stigma may be other reasons.*
- *Multiple presentations for the same chief concern may be red flag for prior misdiagnosis (and, specifically in the case of epistaxis, also happens to be a risk factor for bleeding disorder: https://pubmed.ncbi.nlm.nih.gov/22459034/). Furthermore, evaluations that rely on subjective histories (rather than physical exam findings, lab results, etc.) are more prone to bias.*

Pairs with Group Share in LG (15 min):

3.) Both from the perspectives of clinical reasoning and bias mitigation, what are gaps that you would like to fill when you perform your own H&P. Consider approaches to bias mitigation such as *generating alternative hypotheses, personalizing the patient, deconstructing dominant narratives, direct/constructive feedback, and power sharing, among others (**See Appendix B*** - **Chapter** 5: Deconstructing Racism and Bias in Clinical Medicine of the Textbook of Physical Diagnosis: History and Examination, 8th Edition*)*.

Clinical reasoning:

- *Clarify posterior vs. anterior - bilateral vs. unilateral, bleeding into pharynx, measures taken to stop bleeding, etc.*
- *Is the patient having any signs of anemia?*
- *Is there a history of bleeding elsewhere?*
- *Does the patient have a history of allergic rhinitis?*

Bias mitigation:

- *Assume that the patient is appropriately concerned rather than the dominant narrative that he is overly concerned.* ***Deconstruct this narrative and its potential underlying drivers*** *(e.g. the power differential between immigrant patients and the medical profession influences whose perspectives are deemed valid).*

- *Aim to better understand **WHY** he is so worried (e.g. he has sought medical attention on more than one occasion and have not felt heard; it is possible that he may in fact have a life-threatening condition; or are we missing another concern entirely that is at the root of ongoing presentations, e.g. interpersonal violence resulting in trauma/epistaxis). What does the patient think is going on (some may feel uncomfortable sharing explicitly their worries without being asked)*
- *Gain a better understanding of his prior interactions from his perspective to elucidate prior diagnoses and treatments that have or have not been effective. This includes probing with humility and curiosity regarding the concern for "non-adherence" - **generate alternative hypotheses** and aim to understand rather than making assumptions about the **patient's perspective, motivations, and potential barriers**.*

Back to the case - You return to speak with and examine the patient. You quickly realize that the patient's first language is [Mam] and not Spanish and so he speaks Spanish a bit more slowly. Mam is a Mayan language spoken by half a million people in various regions of Guatemala and Chiapas state of Mexico. A Mam diaspora of thousands exists throughout the United States and Mexico with notable populations in Oakland, California and Washington, D.C. The Mam interpreter is not currently available, but you schedule them to come to the emergency department in a couple hours. In the meantime, you are Spanish-language certified and perform your history in Spanish. You aim first to understand the patient's level of concern. (Pairs - *5 minutes***)**

You: *I'm sorry about all the nosebleeds that you have been having, it seems that you are pretty concerned about it.*

Patient: *Yes, doctor. The bleeding seems to be getting worse, and I don't know what's wrong. It doesn't seem normal!*

You: *I'm glad you're here, so we can get to the bottom of this. Could you tell me more about that? How is the bleeding getting worse?*

Patient: *Well, it started a few months ago and happened about once a week. In the past few days, it has been happening 4 to 5 times per day! It lasts about 10-15 minutes, and there seems to be a lot of blood!*

You: *I see. Anyone in your situation would be concerned—I'm glad you're here so we can think more about it together. Does the bleeding come from one side or both sides?*

Patient: *Both.*

You: *And what do you do to try to make it stop. Many people think they are supposed to squeeze up here [gesturing to the bridge of the nose]. Do you squeeze on the soft part of your nose or up higher?*

Patient: *I do squeeze on the soft part of my nose. But it just keeps on bleeding. The blood goes backwards into my throat, and I feel like I am drowning! I really think I have lost a lot of blood.*

You: *That sounds really scary! Have you had any lightheadedness or feeling of weakness?*

Patient: *I did feel lightheaded and weak today after my nosebleed.*

You: *Any racing of your heart or shortness of breath?*

Patient: *No.*

You: *I see. Thank you. I'm so sorry that you have been going through this and that it's still going on even after all that you've tried. I am so grateful that you are here and am concerned about your bleeding as well. We are going to do some more tests to figure this out. You are in the right place.*

If it's ok, I wanted to also ask about the nasal congestion that you've been experiencing. Could you describe what this feels like?

Patient: *Over the past few months, I have had a lot of congestion in my nose. It feels like my nose is plugged up. Sometimes it's runny, but not much.*

You: *It sounds like this is somewhat new. Have you ever had this issue before these past few months?*

Patient: *Nothing really like this.*

You: *I see. Have you had any itching or watering of your eyes, sneezing, or itching of your nose?*

Patient: *No, mostly just congestion.*

You: *Have you been able to take your medications?*

Patient: *Yes.*

You: *And have the medications that you have been prescribed helped at all?*

Patient: *I haven't really noticed a difference. If anything, the congestion has gotten slowly worse over time.*

You: *I understand. Thank you for taking the time to tell me about your symptoms. I'll do a quick examination and then we'll talk about next steps.*

Clinical Pause and Case Conclusion (Large Group - *10 minutes***):**

1.) Write an updated problem representation.

Many reasonable options, one example:

Adolescent boy (he/him) with uncertain history of allergic rhinitis in context of progressive nasal congestion presenting with acute on chronic, worsening bilateral epistaxis and recent episode of dizziness concerning for posterior bleeding and symptoms of anemia, presently hemodynamically stable.

2.) What might be your diagnostic next steps?
- CBC diff, CMP, PT/PTT, INR, VWD panel / bleeding panel, type and screen (Clinical teaching NOTE: mucosal bleeding is most commonly caused by thrombocytopenia)
- ENT consult, consider hematology consult

Treatment might include: Epistaxis prevention (nasal saline irrigation, Aquaphor to anterior nares bilaterally) and epistaxis treatment (oxymetazoline at bedside to be used prn in addition to anterior compression; floseal coagulant product at bedside)

Clinical Case Conclusion:
Labs are notable for anemia with Hgb 11.3, Hct 32 (MCV 85), normal WBC 5.7, normal plts 299, normal coags (PT 14.1, PTT 30.5, INR 1.0), normal CMP, low normal thrombin time 14.6, normal fibrinogen 347; factor VIII, von Willebrand Factor, Ristocetin Cofactor pending (ultimately normal).

Given finding of anemia (abnormal in an otherwise healthy adolescent boy), ENT consulted and performed bedside scope, which demonstrated the following:
Violaceous mass between the middle turbinate and septum in right nasal cavity and extending superiorly along middle turbinate in left nasal cavity, with associated clot; otherwise unable to advance scope to choana due to obstructive mass.

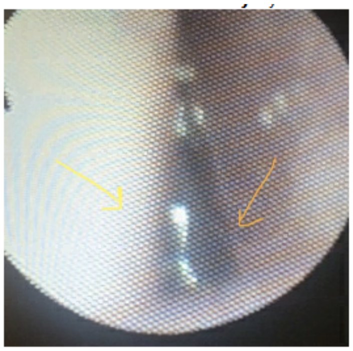 **View of RIGHT nasal cavity - yellow arrow indicates middle turbinate, orange arrow indicates mass.**	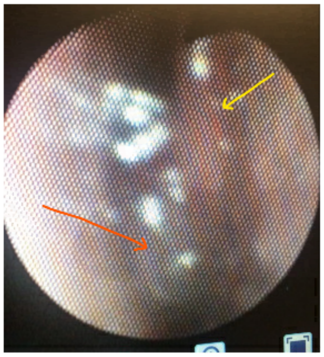 **View of LEFT nasal cavity - yellow arrow indicates head of the inferior turbinate, orange indicates mass seen between the inferior turbinate and septum.**

With concern for a rare but must know cause of posterior epistaxis seen almost exclusively in adolescent cis-gendered boys, **juvenile nasopharyngeal angiofibroma**, vs. malignancy vs. less likely polyposis, ENT recommended CT max/face with and without contrast, which demonstrated a large enhancing nasopharyngeal soft tissue mass with multiple sites of bony erosion and associated intracranial extension.

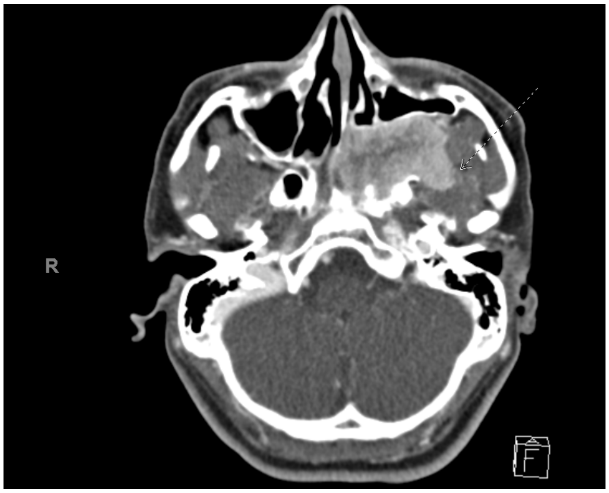

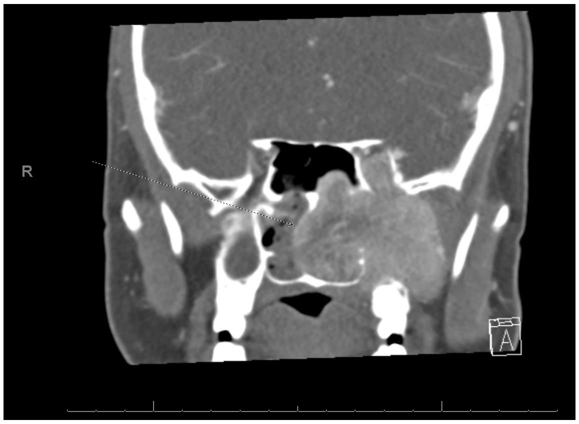

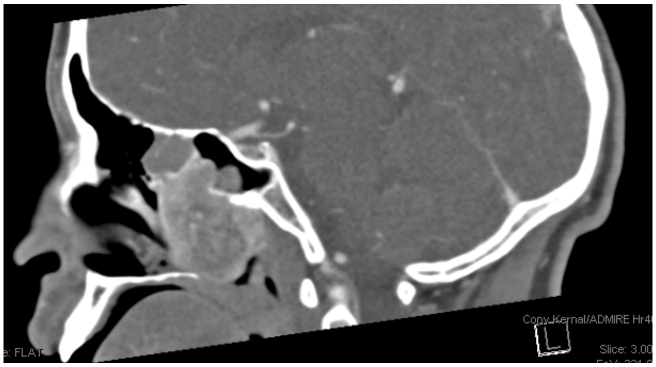

After above CT findings, patient underwent **MR/MRA Brain w/ and w/out contrast** which confirmed a large multispatial enhancing tumor most consistent with **juvenile angiofibroma** with tumor extending into the left foramen rotundum, inferior orbital fissure/orbital apex, likely anterior left cavernous sinus, left nasal passageway and nasopharynx, and left infratemporal fossa with extensive vascular supply. He underwent **IR embolization and subsequently surgical resection** without complication. This diagnosis and hospitalization posed significant financial and social stress on the patient and his sister, who already faced financial and housing insecurities worsened by the COVID pandemic. PCP and outpatient case management were able to connect with a variety of resources.

Systems and Cognitive Bias Reflection (Pairs → LG - *20 minutes***):**

On further review of the patient's chart, you note the following visits prior to presentation to your pediatric emergency department:

August 20 - ***Telephone Visit with PCP clinic NP*** *- first medical visit in US, nasal congestion x 1 month, difficulty breathing through nose, headaches, loss of taste and smell, briefly had attended high school and dropped out with COVID closures → prescribed nasal fluticasone and loratadine, tested for COVID*

September 1 - ***Telephone visit with PCP clinic NP*** *- Mam interpreter, ongoing nasal congestion and small nosebleeds, epistaxis thought perhaps worsened by fluticasone (known compilation), COVID negative → fluticasone discontinued, loratadine switched to cetirizine.*

September 18 - Telephone visit with PCP clinic NP - *ongoing congestion, difficulty sleeping due to congestion, no nosebleeds → trialed systemic (pseudoephedrine) and nasal (oxymetazoline) decongestant, plan for ENT if no improvement.*

September 21 - Telephone visit with PCP - *intent was for in-person visit though scheduled as telephone;* ***ongoing*** *congestion without much improvement despite cetirizine daily → added nasal saline rinses, humidification, Neti Pot with plan for ENT if no improvement, follow-up scheduled for February*

October 8 - First in-person visit, Emergency Department - *now 4 months of ongoing nasal congestion, asking for another medication, swelling of nasal turbinate without bleeding → "has not been on Flonase [fluticasone]," so prescribed this medication and advised to follow-up with PCP.*

October 25 - Second Emergency Department visit - *3 days of epistaxis, right > left nare, longest duration 35 minutes, fluticasone has not been helpful, "not holding direct pressure," dried/crusted blood in bilateral nares without bleeding → advised direct pressure and nasal moisturization with Vaseline, return precautions for prolonged bleeding > 30 minutes.*

October 27 - Visit to your Pediatric Emergency Department *- with epistaxis.*

Have students discuss: What are the **systems**-**level factors** and **cognitive biases** that may have contributed to this patient's prolonged path to diagnosis? Consider both the **strengths** of this system and potential **areas for growth**.

- Inconsistent recognition of need for and availability of **medical interpretation**, particularly Mam. Appointment length does not account for need for interpreter (i.e. LEP patients essentially have half the time of the visit)
- **Telehealth** as facilitator of access, expanded significantly during COVID (evidenced by ability to have multiple visits with PCP office in short time period); however, physical exam limited, particularly if phone visit vs. video visit (? access to technology).
- **Electronic medical records**, while enhancing communication, may also make it easier for inaccuracies and biases to perpetuate over time, even across health care settings.

- Patient was an incredibly perseverant **advocate for himself**, first seeking care at his primary care clinic and then an emergency department and finally an emergency department that happened to have pediatric expertise.
- Highlight how it takes an **expert clinician** with keen **diagnostic reasoning skills** to correct a misdiagnosis influenced by social biases which is why we teach anti-racist clinical skills alongside clinical reasoning.
- **Cognitive Biases (https://www.maimonidesem.org/blog/cognitive-errors -** if time, consider having the students peruse this resource and find types of biases that fit with the case**).** Cognitive biases often occur when the cognitive load of providers is high.
    - **Anchoring** - fixating on specific features of a presentation too early in the diagnostic process and subsequent failure to adjust
    - **Premature closure** - accepting a diagnosis before it has been fully verified
    - **Sutton's slip** - fixation on the most obvious answer
    - **Triage cueing** - a predisposition toward a diagnosis because of a judgment made by a triage provider, whose care may have been brief and early in the process
    - **Zebra retreat** - not willing to pursue a rare diagnosis for a variety of reasons (delay in departmental flow, time intensive workup)

Appendix A: Critical Appraisal of Race in Medical Literature (CARMeL) Tool

Domain	Appraisal Questions
**Internal Validity**	Do the authors clearly define race? If so, how? Is this definition consistent throughout the data collection, analysis and discussion?
	To what extent does this article relay a biologic versus sociopolitical understanding of race?
	To what extent do the authors clearly define how data on race were collected and organized?
	If applicable, were those who analyzed race blinded to the trial interventions?
**External Validity**	To what extent do the options for race collected, reported and analyzed in this paper reflect typical, contemporary racial identities, or the understanding of racial identity with my patient population?
	Are racial categories missing and/or conflated?
**Applicability/Impact**	Are there significant social, political or economic drivers of health that may be obscured by conclusions made in this article?
	In what ways does the use of race in this article contribute to dominant narratives?

**Summary with Recommendation**	**CHOOSE ONE:**<br>*APPLY*: Race is used as a sociopolitical construct with appropriate methods and no significant threats to internal or external validity. The sociopolitical implications are evaluated, and the data is deemed appropriate to apply to patients or populations.<br>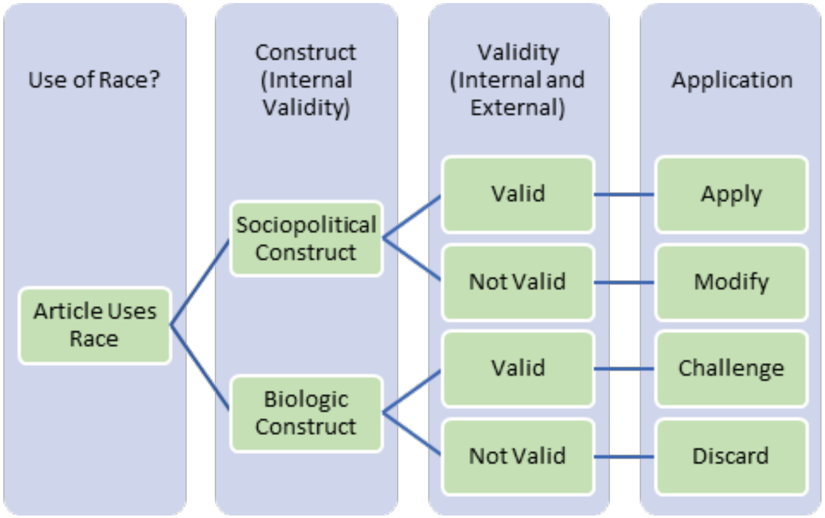<br><br><br>*MODIFY*: Race is used as a sociopolitical construct, but with some threats to internal or external validity. In this case appropriate observations are accepted, but due to methodologic flaws providers will need to modify the way they are applied.<br><br>*CHALLENGE:* Race is likely used as a biologic construct, but with few to little other threats to the internal validity of the study. These studies often yield accurate observations of racial inequities in health but draw inaccurate conclusions regarding causality (i.e. posit racial differences in the prevalence of illness or response to treatment, as opposed to racism and oppression as the cause of the observations). The results of such studies can be used, however the political causes of the observed inequities must be attributed and dismantled. Examples include studies that observe true racial inequities in cardiovascular outcomes but attribute them to biological differences.<br><br>*DISCARD:* Race is used as a biologic construct with significant threats to internal and/or external validity. The results should be challenged, and the conclusions not applied. This includes studies that suggest differential diagnostic tools, treatment algorithms or interventions based on biologic definitions of race, for example studies used to purport racial differences in glomerular filtration rate or expected lung function.

Appendix B: Anti-Racist Clinical Skills/Approaches

Table 1: Sample of Anti-Racist Clinical Skills (from Chapter 5: Deconstructing Racism and Bias in Clinical Medicine of the Textbook of Physical Diagnosis: History and Examination, 8th Edition.)

Skill	Description	Example
**Generate alternative hypotheses**	Goal here is to identify and challenge the label that drives your bias, then identify the real structural forces at play.	Patient with multiple emergency department (ED) visits labeled as a “frequent flyer.” View ED visits as a symptom and seek to identify broader structural barriers to care that force patient into using ED as an access point.
**Personalize the patient**	An active measure to reduce the impact of an identified implicit bias within the clinician triggered by patient name, socioeconomic status, and/or race (identified or presumed). This can also be used universally, with all patients, to reduce the impact of implicit biases of which the clinician may not be aware	Clinician recognizes an implicit bias regarding patients of color and pain medication seeking. The clinician intentionally and purposefully engages in conversation and/or takes a more detailed social history to personalize the patient and reduce the impact of the bias on clinical decision making.
**Deconstruct dominant narratives**	A preparatory measure wherein the clinician seeks to learn about the historical and structural drivers of dominant narratives that support his or her implicit biases.	Clinicians learn the historical relationship between their community and medicine to better interpret clinical observations. A patient complaining about long wait times for appointments and never seeing the same doctor twice while receiving care in a resident clinic should be viewed in the context of historic and contemporary segregation of care—once explicitly by race, now indirectly race via insurance status. Some patients’ mistrust of the medical system should be viewed in the context of historic and contemporary experimentation upon individuals and communities of color.
**Framing**	Grounded in the well-documented cognitive theory of framing, in which the way a patient is presented can impact the clinical reasoning of the listener or reader. Clinicians can use this as an anti-racist skill by reframing a patient to call attention to structural drivers of an observed health outcome. This skill is also used to counter dominant narratives.	A black mother who voices concerns about the care her child received in the hospital is labeled as disruptive or angry. The clinician highlights mother’s advocacy and uses language such as “invested in her child’s health” and “advocate for her child” when describing the family to colleagues.
